# Comparative assessment of heel rise detection for consistent gait phase separation

**DOI:** 10.1016/j.heliyon.2024.e33546

**Published:** 2024-06-24

**Authors:** Mikko Salminen, Jarmo Perttunen, Janne Avela, Antti Vehkaoja

**Affiliations:** a Faculty of Medicine and Health Technology, Tampere University, Korkeakoulunkatu 3, 33720, Tampere, Finland; b Faculty of Sports and Health Sciences, Jyväskylä University, Seminaarinkatu 15, 40014, Jyväskylä, Finland

**Keywords:** Gait analysis, Shank angular velocity, Inertial measurement unit (IMU), Heel-off, Optical motion capture, Event detection

## Abstract

**Background:**

Accurate identification of gait events is crucial to reliable gait analysis. Heel rise, a key event marking the transition from mid-stance to terminal stance, poses challenges in precise detection due to its gradual nature. This leads to variability in accuracy across studies utilizing diverse measuring techniques.

**Research question:**

How do different HR detection methods compare when assessed against the underlying heel motion pattern and visual detection across varying speed, footwear conditions, and individuals?

**Methods:**

Leveraging data from over 10,000 strides in diverse scenarios with 15 healthy subjects, we evaluated methods based on measurements from optical motion capture (OMC), force plates, and shank-mounted inertial measurement units (IMUs). The evaluation of these methods included an assessment of their precision and consistency with the heel marker's motion pattern and agreement with visually detected heel rise.

**Results:**

OMC-based heel rise detection methods, utilizing the heel marker's vertical acceleration and jerk, consistently identified the same point in the heel motion pattern, outperforming velocity-based methods and our new position-based method resembling traditional footswitch-based heel rise detection. Variability in velocity and position-based methods derives from subtle heel rise variations after mid-stance, exhibiting individual differences. Our proposed IMU-based methods show promise by closely matching OMC-based accuracy.

**Significance:**

The results have significant implications for gait analysis, providing insights into heel rise event detection's complexities. Accurate HR identification is crucial for gait phase separation, and our findings, especially with the robust heel marker's jerk-based method, enhance precision and consistency across walking conditions. Moreover, our successful development and validation of IMU-based algorithm offer cost-effective and mobile alternative for HR detection, expanding their potential use in comprehensive gait analysis.

## Highlights

• We assess motion capture, force plate, and IMU data to identify the most precise heel rise detection method.
• Subtle rising of the heel before the actual lift-off causes variability in heel rise detection.
• Heel marker's acceleration and jerk-based methods are in high agreement with visual detection.
• Novel IMU-based methods closely mirror the performance of acceleration and jerk-based methods.
• The proposed methods improve heel rise detection accuracy for gait analysis.

## 1 Introduction

Understanding human locomotion is essential for a wide range of applications in healthcare, sports, and biomechanics. Gait analysis is a fundamental tool in these applications.

One widely acknowledged method for gait analysis is marker-based optical motion capture (OMC). When combined with force plates, OMC enables in-depth examination of gait kinematics and kinetics, and is widely considered the gold standard in gait analysis [1]. However, the limitations of these lab-based tools i.e. the requirement of a specific location, high cost, requirement of skilled operator and limitation in the investigation area necessitate consideration of additional field-friendly approaches for use in healthcare settings and homes. In response to this need, inertial measurement units (IMU) have emerged as a portable and cost-effective substitute for traditional gait analysis tools [2]. Among the various methods of measuring gait using IMU's, the utilization of a single shank-mounted sensor has gained prominence [3].

At the core of gait analysis lies the precise detection of key gait events, enabling a detailed temporal examination of the gait cycle. The most significant events, initial contact and toe-off, divide the cycle into stance and swing phases. Opposite foot initial contact, opposite foot toe-off, heel rise, feet adjacent, and tibia vertical further divide the cycle into seven sub-phases [4]. Analyzing a person's walking pattern through these phases and their relative durations allows for a more direct identification of the functional significance of different motions, which is particularly important for interpreting the functional effects of disabilities [5].

Initial contact, toe-off, and swing-related events have well-established and accurate detection methods when using OMC, force plates, and IMU's [[6], [7], [8], [9], [10], [11]]. However, the challenge arises with heel rise (HR), marking the transition from mid-stance to terminal stance phases. This event exhibits considerable variation with walking speed and individual differences [4]. HR is defined as the moment when “the heel rises from the surface as measured with footswitches” [5] or “the heel begins to lift from the walking surface” [4].

During heel rise, the center of mass (COM) advances over the forefoot, the swinging limb is anterior to the COM and its inertia with the falling body weight act as a propelling force. The foot segment between the ankle and forefoot serves as a lever aiming to preserve leg height. As the calf muscles preserve the forefoot rocker, the heel begins its rapid rise [5]. Accurate detection of HR is crucial, as an early or delayed HR can indicate excessive or insufficient motion in joints, potentially signaling muscle weaknesses or lack of control [5].

However, accurately detecting HR presents a substantial challenge due to its gradual nature, resembling a phase rather than a distinct event. The graduality is further influenced by the elasticity of the shoe [12]. Consequently, the challenges have resulted in variability in HR detection across various studies that employ different measurement techniques, including visual detection, force plates, footswitches, accelerometers, gyroscopes, and OMC [7,10,[13], [14], [15], [16], [17], [18], [19], [20], [21], [22]]. While the preferred pressure-sensing techniques like footswitches provide valuable timing information, they have limitations in offering comprehensive insights into HR and are prone to inaccuracies caused by sensor positioning [17,19].

To address these challenges, our study employed a comprehensive approach, focusing on analyzing HR and the associated heel motion pattern using gold standard techniques (OMC and force plates), supplemented by visual detection. Our prospectively collected dataset comprises 10,584 gait cycles from healthy young adults walking at various speeds, both with shoes and barefoot, and with varying heel heights. Our primary objective was to establish a well-proven HR detection method for gait phase separation. Additionally, we aimed to introduce and validate a novel shank-attached IMU-based approach to fill the identified gap in Ref. [10].

## 2 Methods

### 2.1 Experimental set-up

Fifteen healthy volunteers, comprising 5 males and 10 females, with a mean age of 23.7 years (±3.5 years SD), a mean height of 170 cm (±10 cm), and a mean weight of 70 kg (±14 kg), participated in this study. Measurements were performed in a biomechanics laboratory along a 12-m walkway. Participants wore their own shoes adept for walking (indoor sports shoes or similar). Prior to participation, all subjects provided signed informed consent. The study adhered to the Declaration of Helsinki and received ethical statement (199/13.00.04.00/2022).

The gait of each subject was simultaneously measured using three gait measurement technologies: two shank-worn Vicon Blue Trident IMUs, two AMTI (Advanced Mechanical Technology Inc.) force plates, and a Vicon OMC system (Vicon Motion Systems Ltd, Oxfordshire, UK). In OMC measurements, a Plug-in-Gait lower body model with 16 markers was employed. The IMUs were positioned on the lateral side of the shank, just above the lateral malleolus similarly as in Ref. [11]. To achieve the correct alignment, the sensor's vertical axis was parallel to the longitudinal axis of the shank segment, while the anteroposterior axis aligned with the walking direction, following the imaginary line connecting the back of the heel and the second metatarsal head.

The study encompassed nine walking scenarios, including slow (4 km/h), normal (5 km/h), and fast (6.5 km/h) walking in both shod and barefoot conditions. Additionally, subjects walked with different heel heights (2 mm, 6 mm, and 10 mm) while wearing shoes at normal speed. Walking speed was controlled with photocell timing.

The synchronization of data from different measuring technologies, and OMC data preprocessing, were both done using Vicon Nexus 2.14 motion capture software. Sample rates for force plates, OMC and IMU's were 1125 Hz, 187.5 Hz and 225 Hz, respectively, which were then linearly resampled to match force plate sample rate.

### 2.2 Data analysis

All post-processing and analysis were carried out within the Python 3.10 programming environment.

Initially, gait cycles were identified by detecting initial contacts, defined as the first local minima following the distinctive mid-swing peak in the mediolateral shank angular velocity signal from the IMU sensor [6]. Of the 24,519 recorded strides, a balanced number per participant, foot, and scenario was selected for analysis (Table 1). This subset of 10,584 strides included those that most closely matched the target speed for each scenario based on OMC measurements.

**Table 1:** Stride counts and key gait parameters for fast, normal, and slow walking speeds in various shoe scenarios.

	Shoe scenario	All	BAREFOOT			SHOE			2 mm	6 mm	10 mm
	Speed	Total	fast	normal	slow	fast	normal	slow	normal	normal	normal
**STRIDE COUNT**	Total (n)	10584	828*	1800	900	836**	1800	900	1200	1200	1120***
**STRIDE COUNT PER SUBJECT'S LEG**	Mean (n)		29.6*	60	30	29.9**	60	30	40	40	40***
**STRIDE TIME**	Mean (s)		0.92	1.02	1.14	0.95	1.05	1.17	1.05	1.05	1.06
	*SD of leg means (s)*		*0.06*	*0.05*	*0.06*	*0.05*	*0.06*	*0.07*	*0.05*	*0.06*	*0.06*
**STRIDE LENGTH**	Mean (m)		1.64	1.41	1.27	1.71	1.45	1.31	1.45	1.45	1.46
	*SD of leg means (m)*		*0.09*	*0.07*	*0.06*	*0.10*	*0.08*	*0.07*	*0.07*	*0.08*	*0.08*
**STRIDE VELOCITY**	Mean (m/s)		1.78	1.38	1.11	1.79	1.38	1.12	1.38	1.38	1.38
	*SD of leg means (m/s)*		*0.02*	*0.00*	*0.01*	*0.03*	*0.00*	*0.01*	*0.01*	*0.00*	*0.01*
	*Mean leg-specific SD (m/s)*		*0.04*	*0.02*	*0.03*	*0.04*	*0.02*	*0.03*	*0.03*	*0.01*	*0.03*

Note: *S15, **S05, and ***S06 data are missing due to technical issues and are not included in the mean stride count per subject's leg.

Presented data analysis focuses on evaluating the accuracy of HR detection by comparing the timing and vertical position of the heel at the moment of detection across various methods. More specifically, we examined how these detections aligned with the motion pattern of the heel rise in diverse scenarios. To verify the normality of the data, a Shapiro-Wilk test was performed (Table S1). Additionally, we conducted an in-depth study of the variability in heel rise event occurrence, both within a subject and scenario, and between subjects. Given our highly strict protocol and the minimal variability of walking speed in the strides included in the dataset, we anticipated low variability of the detection point for methods that detect HR with high precision. For these analyses, the gait cycle was time normalized to 0–100 % of stride time.

Finally, visual detection, as in Refs. [[13], [14], [15]] was used to detect HR. The detection was based on the vertical motion of the heel marker and was conducted using a tool and data accessible in Ref. [23]. This analysis focused on a subset of data, comprising 6 randomly selected strides for normal speed and 3 for slow and fast speeds for each leg of all subjects in both shod and barefoot walking conditions. In total, the subset included 701 strides. Accuracy of the methods were evaluated by measuring true error and agreement against visual detection in milliseconds. Agreement was evaluated with Bland-Altman plots [24] and intra-class correlation coefficients (ICC). ICC scores and their 95 % confident intervals were calculated based on a 2-way mixed-effects model [25].

### 2.3 Heel rise detection methods

We detected HR using three signals: the vertical motion of the heel marker, the anteroposterior force from the force plate (Fy), and the longitudinal acceleration from the IMU, which was aligned with the shank segment's longitudinal axis. The first two signals were first filtered with a 2nd order zero-phase Butterworth lowpass filter with a 10 Hz cutoff frequency using forward-backward filtering. However, for the accelerometer signal, a similar filter was used with a 6 Hz cutoff frequency. Heel marker's vertical velocity, acceleration, and jerk were calculated as the first, second, and third time derivatives of the vertical position, respectively. For jerk calculation, the heel marker's acceleration signal was filtered a second time with a 10 Hz cutoff frequency.

**Fig. 1:**
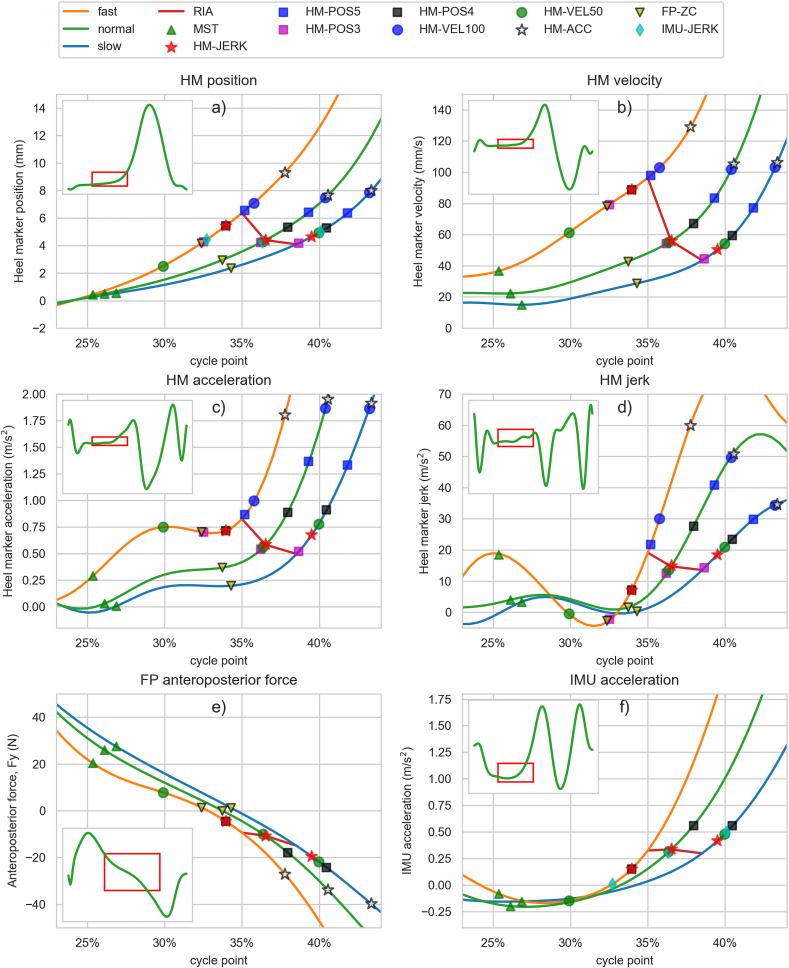
Mean signal plots during heel rise for fast (orange), normal (green) and slow (blue) walking in shoes, along with mean HR detection points of different methods: a) Heel marker vertical position relative to position at MST, b) velocity, c) acceleration, d) jerk (velocity of acceleration), e) force plate anteroposterior force (Fy), f) IMU longitudinal acceleration (gravity being subtracted). Only the most relevant method detections are visible for each signal. The red line indicates the instant of rapid increase in acceleration (RIA), which we have visually detected based on these mean velocity and acceleration plot shapes. Smaller subplots depict signal shape during the entire gait cycle. (For interpretation of the references to colour in this figure legend, the reader is referred to the Web version of this article.)

To detect HR, we first identified the mid-stance event (MST), occurring during the mid-stance phase when the toe marker of the swinging foot surpassed the heel marker of the stance foot [4]. We chose MST as the reference point for the heel's vertical position because we assumed that heel rise had not yet occurred, and the weight distribution on the foot's sole was relatively even at this point. After detecting the MST, following methods were used for detecting the HR:

*Position-based methods (HM-POS):* HR is detected when the vertical position of the heel marker exceeds the position at MST by 5, 4, or 3 mm after MST (Fig. 1a), referred to as HM-POS5, HM-POS4, and HM-POS3, respectively. MST is used as the reference to mitigate the impact of marker placement and shoe sole thickness. The method is designed to mimic pressure-based HR detection, where the threshold is the height at which the pressure sensor elevates from the surface.

*Velocity-based method (HM-VEL):* HR is detected when the vertical velocity of the heel marker exceeds 100 mm/s, 80 mm/s or 50 mm/s after MST (Fig. 1b), referred to as HM-VEL100, HM-VEL80, and HM-VEL50, respectively. These methods have been previously used in Refs. [14,16,20,21].

*Acceleration-based method (HM-ACC):* HR is detected when the vertical acceleration of the heel marker exceeds 1.9 m/s^2^ after MST (Fig. 1c) [10].

*Jerk-based method (HM-JERK):* HR is detected when the vertical jerk of the heel marker exceeds 15 m/s^3^ the last time before the acceleration exceeds 1.9 m/s^2^ after MST to identify the moment when acceleration begins to increase more rapidly (Fig. 1d).

*Force plate-based method (FP-ZC):* HR is detected as the zero-crossing of the force plate's anteroposterior signal during mid-stance phase, when the force turns from decelerating into accelerating (Fig. 1e) [4,22].

*IMU's acceleration-based method (IMU-ACC):* HR is detected as the moment when IMU's longitudinal accelerometer data, where gravity is subtracted, exceeds a threshold of 1 m/s^2^ after MST (Fig. 1f). The threshold was set to the lowest possible level that would still avoid premature detections due to shank motion before heel rise, even at fast speeds.

*IMU's jerk-based method (IMU-JERK):* HR is detected as the last time before the shank's longitudinal accelerations 1 m/s^2^ (IMU-ACC) threshold, when jerk is below 12.5 m/s^3^ to identify the moment when acceleration begins to increase more rapidly. The threshold was set to the lowest possible level that would still detect the beginning of rapid acceleration increase, leading to full forefoot rocker motion.

*Visual detection:* HR is detected visually using the heel marker's vertical position, velocity, and acceleration data graphs (Fig. S4), as the moment when the velocity distinctly demonstrates an accelerating increase, signifying the initiation of the full forefoot rocker motion [23].

## 3 Results

In Fig. 1, we display the alignment between the detected HR event options and the corresponding heel motion patterns in different scenarios. From Fig. 1a we can see that it is challenging to precisely pinpoint the moment of the actual HR from the heel marker position, given the gradual nature of the motion. However, by examining velocity, acceleration, and jerk of the positional change, we can amplify the heel motion pattern and better identify key events throughout its progression.

When examining velocity (Fig. 1b), we observe a gradual increase after MST, followed by a more rapid increase in acceleration (RIA) that initiates the distinct heel rise toward a full forefoot rocker motion. The RIA point becomes more evident in the mean acceleration plot (Fig. 1c). The RIA point was visually identified based on the shape of mean velocity and acceleration plots and marked with a red line in Fig. 1a–f. Based on this, the RIA occurs at approximately 35 %, 36.5 %, and 38.5 % of the gait cycle in shod walking and 33.5 %, 35 %, and 37 % in barefoot walking, corresponding to fast, normal, and slow walking speeds, respectively. The HM-JERK, IMU-JERK, HM-POS3, and HM-VEL50 methods detect HR in shod walking closest to the RIA in terms of means. However, in barefoot conditions, the HM-POS and HM-VEL methods tend to detect HR earlier, with optimal thresholds at 4 mm and 80 mm/s, respectively (Table 2, Fig. S1).

**Table 2:** Comparison of HR detection methods in different shoe and speed scenarios. This table presents mean cycle points (cp), heel marker vertical positions relative to MST (pos), and their variability for each method. ‘SD of leg means' represents variability between individuals, while ‘Mean leg-specific SD’ represents mean SD within a leg, indicating within-session variability.

	Shoe scenario Speed	All		BAREFOOT						SHOE						2 mm	6 mm	10 mm
				fast		normal		slow		fast		normal		slow		normal	normal	normal
		cp	pos (mm)	cp	pos (mm)	cp	pos (mm)	cp	pos (mm)	cp	pos (mm)	cp	pos (mm)	cp	pos (mm)	cp	cp	cp
**MST**	Mean	26.1 %	0.0	25.1 %	0.0	25.8 %	0.0	26.1 %	0.0	25.4 %	0.0	26.1 %	0.0	26.8 %	0.0	26.3 %	26.4 %	26.5 %
	*SD of leg means*	*0*.*7 %*	*-*	*0*.*7 %*	*-*	*0*.*7 %*	*-*	*0*.*6 %*	*-*	*0*.*7 %*	*-*	*0*.*7 %*	*-*	*0*.*7 %*	*-*	*0*.*7 %*	*0*.*6 %*	*0*.*7 %*
	*Mean leg-specific SD*	*0*.*6 %*	*-*	*0*.*7 %*	*-*	*0*.*6 %*	*-*	*0*.*6 %*	*-*	*0*.*6 %*	*-*	*0*.*5 %*	*-*	*0*.*6 %*	*-*	*0*.*5 %*	*0*.*5 %*	*0*.*6 %*
**HM-POS 3**	Mean	35.6 %	3.0	30.6 %	3.0	33.3 %	3.0	35.3 %	3.0	32.5 %	3.0	36.2 %	3.0	38.8 %	3.0	36.9 %	38.1 %	39.0 %
	*SD of leg means*	*2*.*2 %*	*-*	*1*.*3 %*	*-*	*1*.*6 %*	*-*	*1*.*8 %*	*-*	*2*.*0 %*	*-*	*2*.*6 %*	*-*	*2*.*7 %*	*-*	*2*.*8 %*	*2*.*5 %*	*2*.*4 %*
	*Mean leg-specific SD*	*1*.*8 %*	*-*	*1*.*3 %*	*-*	*1*.*5 %*	*-*	*1*.*8 %*	*-*	*1*.*7 %*	*-*	*2*.*0 %*	*-*	*2*.*2 %*	*-*	*1*.*9 %*	*1*.*9 %*	*1*.*9 %*
**HM-POS 4**	Mean	37.3 %	4.0	31.8 %	4.0	34.8 %	4.0	36.9 %	4.0	33.9 %	4.0	38.0 %	4.0	40.6 %	4.0	38.7 %	39.9 %	40.8 %
	*SD of leg means*	*2*.*2 %*	*-*	*1*.*4 %*	*-*	*1*.*7 %*	*-*	*1*.*8 %*	*-*	*2*.*2 %*	*-*	*2*.*6 %*	*-*	*2*.*7 %*	*-*	*2*.*7 %*	*2*.*4 %*	*2*.*3 %*
	*Mean leg-specific SD*	*1*.*8 %*	*-*	*1*.*4 %*	*-*	*1*.*5 %*	*-*	*1*.*8 %*	*-*	*1*.*9 %*	*-*	*2*.*0 %*	*-*	*2*.*1 %*	*-*	*1*.*8 %*	*1*.*8 %*	*1*.*8 %*
**HM-POS 5**	Mean	38.5 %	5.0	32.8 %	5.0	36.1 %	5.0	38.2 %	5.0	35.1 %	5.0	39.3 %	5.0	41.9 %	5.0	40.0 %	41.2 %	42.0 %
	*SD of leg means*	*2*.*2 %*	*-*	*1*.*5 %*	*-*	*1*.*8 %*	*-*	*1*.*7 %*	*-*	*2*.*3 %*	*-*	*2*.*6 %*	*-*	*2*.*6 %*	*-*	*2*.*6 %*	*2*.*3 %*	*2*.*2 %*
	*Mean leg-specific SD*	*1*.*7 %*	*-*	*1*.*4 %*	*-*	*1*.*5 %*	*-*	*1*.*8 %*	*-*	*2*.*0 %*	*-*	*1*.*9 %*	*-*	*2*.*0 %*	*-*	*1*.*8 %*	*1*.*7 %*	*1*.*7 %*
**HM-VEL 50**	Mean	34.9 %	2.1	25.8 %	−0.2	31.5 %	1.9	35.2 %	3.0	29.8 %	1.3	36.4 %	3.0	40.0 %	3.6	37.2 %	38.7 %	39.8 %
	*SD of leg means*	*2*.*7 %*	*0*.*9*	*2*.*2 %*	*1*.*0*	*2*.*6 %*	*0*.*7*	*2*.*1 %*	*0*.*5*	*3*.*6 %*	*1*.*3*	*3*.*3 %*	*0*.*8*	*2*.*9 %*	*0*.*7*	*3*.*1 %*	*2*.*5 %*	*2*.*2 %*
	*Mean leg-specific SD*	*2*.*5 %*	*1*.*1*	*2*.*5 %*	*1*.*5*	*2*.*8 %*	*1*.*2*	*2*.*5 %*	*0*.*9*	*3*.*1 %*	*1*.*2*	*2*.*7 %*	*1*.*0*	*2*.*2 %*	*0*.*8*	*2*.*4 %*	*2*.*0 %*	*1*.*8 %*
**HM-VEL 80**	Mean	38.0 %	4.5	30.0 %	2.3	36.0 %	4.9	38.9 %	5.6	33.9 %	3.8	39.3 %	5.0	42.3 %	5.3	39.8 %	40.8 %	41.5 %
	*SD of leg means*	*2*.*2 %*	*1*.*0*	*2*.*5 %*	*1*.*2*	*2*.*3 %*	*1*.*0*	*1*.*7 %*	*0*.*9*	*3*.*0 %*	*1*.*0*	*2*.*5 %*	*0*.*9*	*2*.*0 %*	*1*.*0*	*2*.*4 %*	*2*.*0 %*	*1*.*9 %*
	*Mean leg-specific SD*	*1*.*9 %*	*0*.*6*	*2*.*5 %*	*1*.*3*	*1*.*8 %*	*0*.*3*	*1*.*5 %*	*0*.*3*	*3*.*0 %*	*1*.*0*	*1*.*9 %*	*0*.*4*	*1*.*7 %*	*0*.*5*	*1*.*7 %*	*1*.*4 %*	*1*.*3 %*
**HM-VEL 100**	Mean	39.3 %	5.8	32.0 %	3.9	37.5 %	6.3	40.3 %	7.0	35.6 %	5.4	40.4 %	6.0	43.2 %	6.2	40.7 %	41.5 %	42.1 %
	*SD of leg means*	*2*.*0 %*	*1*.*1*	*2*.*4 %*	*1*.*2*	*2*.*2 %*	*1*.*1*	*1*.*5 %*	*1*.*0*	*2*.*7 %*	*1*.*0*	*2*.*2 %*	*1*.*1*	*1*.*8 %*	*1*.*2*	*2*.*0 %*	*1*.*8 %*	*1*.*8 %*
	*Mean leg-specific SD*	*1*.*6 %*	*1*.*2*	*2*.*4 %*	*1*.*7*	*1*.*5 %*	*0*.*9*	*1*.*3 %*	*0*.*9*	*2*.*7 %*	*1*.*7*	*1*.*5 %*	*1*.*0*	*1*.*5 %*	*1*.*0*	*1*.*4 %*	*1*.*1 %*	*1*.*1 %*
**HM-ACC**	Mean	40.1 %	7.8	35.5 %	8.6	38.8 %	7.9	41.6 %	9.0	37.5 %	7.9	40.6 %	6.6	43.4 %	7.0	40.7 %	41.0 %	41.4 %
	*SD of leg means*	*1*.*6 %*	*2*.*9*	*2*.*8 %*	*3*.*7*	*1*.*4 %*	*2*.*0*	*1*.*3 %*	*2*.*2*	*2*.*3 %*	*3*.*2*	*1*.*3 %*	*2*.*7*	*1*.*4 %*	*3*.*3*	*1*.*2 %*	*1*.*2 %*	*1*.*1 %*
	*Mean leg-specific SD*	*1*.*1 %*	*2*.*5*	*1*.*6 %*	*3*.*4*	*1*.*0 %*	*1*.*9*	*1*.*1 %*	*2*.*3*	*1*.*2 %*	*2*.*9*	*1*.*0 %*	*1*.*9*	*1*.*2 %*	*2*.*3*	*1*.*0 %*	*0*.*9 %*	*0*.*9 %*
**HM-JERK**	Mean	36.1 %	4.6	32.2 %	5.7	34.5 %	4.3	37.3 %	4.8	33.8 %	5.0	36.6 %	3.9	39.5 %	4.3	36.7 %	37.0 %	37.5 %
	*SD of leg means*	*1*.*6 %*	*2*.*2*	*2*.*7 %*	*2*.*9*	*1*.*6 %*	*1*.*6*	*1*.*4 %*	*1*.*6*	*2*.*4 %*	*2*.*5*	*1*.*3 %*	*2*.*0*	*1*.*6 %*	*2*.*5*	*1*.*2 %*	*1*.*2 %*	*1*.*2 %*
	*Mean leg-specific SD*	*1*.*6 %*	*2*.*2*	*2*.*1 %*	*3*.*6*	*1*.*7 %*	*1*.*7*	*1*.*6 %*	*1*.*9*	*1*.*6 %*	*2*.*4*	*1*.*5 %*	*1*.*5*	*1*.*9 %*	*2*.*1*	*1*.*5 %*	*1*.*3 %*	*1*.*2 %*
**IMU-ACC**	Mean	39.9 %	7.6	35.6 %	9.0	38.7 %	8.0	41.0 %	8.2	36.9 %	7.5	40.5 %	6.5	43.0 %	6.4	40.7 %	40.9 %	41.3 %
	*SD of leg means*	*1*.*8 %*	*2*.*5*	*2*.*7 %*	*3*.*0*	*1*.*9 %*	*2*.*0*	*1*.*6 %*	*2*.*0*	*2*.*2 %*	*3*.*4*	*1*.*5 %*	*2*.*0*	*1*.*4 %*	*2*.*6*	*1*.*6 %*	*1*.*7 %*	*1*.*6 %*
	*Mean leg-specific SD*	*1*.*5 %*	*2*.*6*	*1*.*9 %*	*3*.*8*	*1*.*7 %*	*2*.*3*	*1*.*5 %*	*2*.*3*	*1*.*5 %*	*2*.*9*	*1*.*4 %*	*2*.*2*	*1*.*4 %*	*2*.*1*	*1*.*3 %*	*1*.*3 %*	*1*.*3 %*
**IMU-JERK**	Mean	36.0 %	4.5	31.5 %	4.8	34.7 %	4.4	37.9 %	5.4	32.6 %	4.2	36.4 %	3.8	40.2 %	4.5	36.6 %	36.7 %	37.1 %
	*SD of leg means*	*2*.*0 %*	*2*.*2*	*2*.*7 %*	*2*.*5*	*2*.*1 %*	*1*.*6*	*1*.*9 %*	*2*.*0*	*2*.*3 %*	*2*.*7*	*1*.*9 %*	*2*.*0*	*1*.*7 %*	*2*.*4*	*1*.*9 %*	*1*.*7 %*	*1*.*6 %*
	*Mean leg-specific SD*	*1*.*7 %*	*2*.*1*	*2*.*0 %*	*3*.*0*	*1*.*7 %*	*1*.*7*	*1*.*8 %*	*2*.*2*	*1*.*4 %*	*2*.*1*	*1*.*9 %*	*1*.*8*	*1*.*5 %*	*2*.*0*	*1*.*6 %*	*1*.*7 %*	*1*.*5 %*
**FP-ZC**	Mean	33.7 %	3.5	32.5 %	5.1	34.1 %	3.9	34.4 %	3.2	32.3 %	3.8	33.7 %	2.6	34.3 %	2.1	33.9 %	33.9 %	34.2 %
	*SD of leg means*	*2*.*0 %*	*1*.*9*	*2*.*1 %*	*2*.*3*	*1*.*8 %*	*1*.*5*	*2*.*3 %*	*2*.*1*	*2*.*0 %*	*2*.*0*	*1*.*9 %*	*1*.*6*	*2*.*1 %*	*1*.*9*	*1*.*8 %*	*1*.*8 %*	*1*.*9 %*
	*Mean leg-specific SD*	*2*.*0 %*	*1*.*6*	*2*.*3 %*	*2*.*3*	*2*.*0 %*	*1*.*7*	*2*.*0 %*	*1*.*4*	*2*.*3 %*	*1*.*8*	*1*.*9 %*	*1*.*4*	*2*.*0 %*	*1*.*2*	*1*.*5 %*	*1*.*9 %*	*1*.*8 %*

The HR detection methods were also evaluated using effect size analysis (Cohen's d) (Table S2). Results revealed strong similarity between acceleration-based methods HM-ACC and IMU-ACC (0.16), and jerk-based methods HM-JERK and IMU-JERK (0.24). Interestingly, HM-POS4 also showed notable similarity with jerk-based methods, exhibiting effect sizes of 0.25 and 0.28 compared to HM-JERK and IMU-JERK, respectively. The methods HM-ACC, HM-JERK, IMU-ACC, HM-VEL100, and IMU-JERK exhibited the lowest variability in HR event gait cycle point between (SD = 1.6 %–2.0 %) and within scenarios (SD = 1.1 %–1.7 %) (Table 2). Notably, the HM-POS methods showed low variability in barefoot scenarios (SD = 1.3 %–1.8 %) but significantly higher variability when walking in shoes (SD = 2.0 %–2.7 %).

**Fig. 2:**
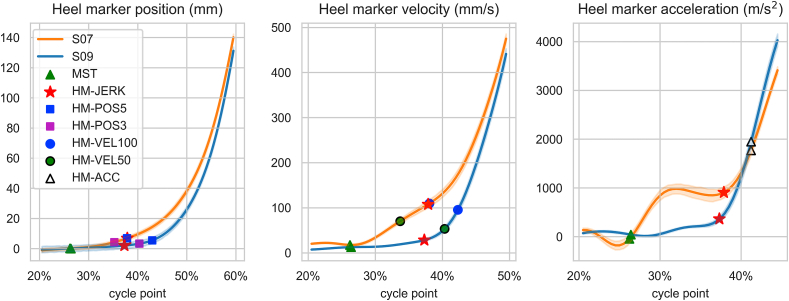
Mean heel marker position, velocity, and acceleration including HR events detected using different methods. The figure illustrates distinct heel rise strategies for subject S07 (orange) and S09 (blue), representing extremes within the subject group. Shaded areas indicate 95 % confidence intervals of the mean. Data is from walking in shoes at a normal speed. (For interpretation of the references to colour in this figure legend, the reader is referred to the Web version of this article.)

A significant factor affecting HR detection is the subtle heel rising between MST and RIA, termed “pre-rising.” Notably, there is considerable variability in pre-rising among subjects, as illustrated in Fig. 2, showcasing the heel marker motion patterns of two subjects with different heel rise strategies. Approximately half of the participants follow a similar heel rise strategy to S09, while the other half displays a pronounced pre-rising before the rapid acceleration, akin to S07 (Fig. S2).

As shown in Fig. 2 and Fig. S6, pre-rising introduces significant variability in HR detection, affecting the timing and alignment of position and velocity-based methods with the heel motion pattern. It also impacts the vertical position of the heel marker in acceleration and jerk-based methods (Table 2). However, when assessing vertical position consistency across scenarios, HM-JERK and IMU-JERK methods demonstrate minimal variability, averaging 4.6 ± 0.6 mm and 4.5 ± 0.5 mm, respectively. In contrast, other methods show means ranging from 2.1 mm to 7.8 mm and SDs from 0.9 mm to 1.4 mm (Table 2, Fig. S3).

In a detailed analysis, we visually detected the RIA point in individual strides. Individual detections can be reviewed with the tool and data provided in Ref. [23]. Additionally, Fig. S4 in the supplementary material presents the mean detection points on position, velocity, and acceleration plots.

**Fig. 3:**
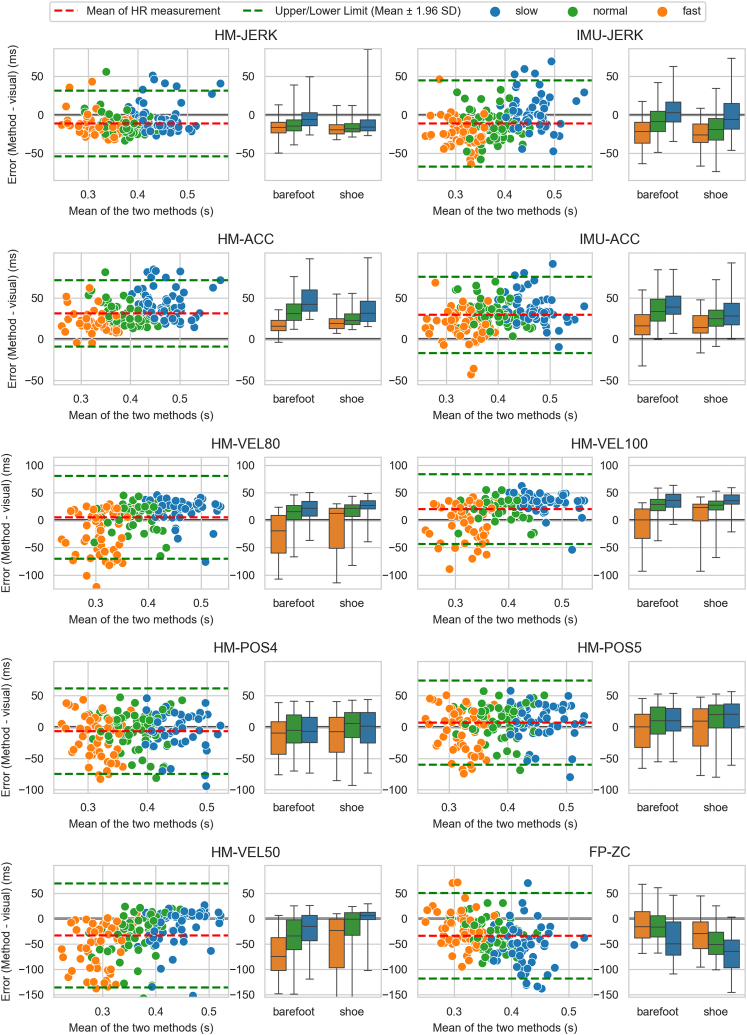
Agreement between HR detection methods and visual detection. Boxplots depict the IQR, and whiskers represent the 95 % confidence interval. The dataset comprises 701 force plate strides evenly distributed across subjects and scenarios.

The comparison between visually detected HR and algorithm-based HR detection methods highlighted the exceptional performance of HM-JERK and IMU-JERK, evidenced by high absolute agreement (ICC: 0.91 and 0.88) and the lowest mean absolute errors (0.19 and 0.24 ms) with mean errors of −11 ± 22 and −11 ± 29 ms (Table 3). When looking at consistency, the ICC demonstrated equally high agreement between visual detection and acceleration-based methods: HM-ACC = 0.94, HM-JERK = 0.94, IMU-ACC = 0.93, and IMU-JERK = 0.90. (Table 3). Agreement between visual detection and methods is visualized in Fig. 3.

**Table 3:** Comparison of visually detected HR and other detection methods. The table presents the mean detection point, difference between visual and method detection (error), the limits of agreement and mean absolute error. Multiple ICC model scores are also presented to show the agreement between the measures.

HR detection method	Mean HR (SD) [s]	Method error (SD) [ms]	Error 95 % Limits of agreement [ms]	Mean absolute error [ms]	*ICC (absolute error,* single rating)	*ICC (absolute error, average values)*	*ICC (consistency,* single rating)	*ICC (consistency, average values)*
**HM-ACC**	0.41 (0.06)	31 (21)	[-9, 72]	32	0.84 [0.02, 0.95]	0.91 [0.04, 0.97]	0.94 [0.94, 0.95]	0.97 [0.97, 0.98]
**HM-JERK**	0.37 (0.06)	−11 (22)	[-54, 31]	19	0.92 [0.85, 0.95]	0.96 [0.92, 0.97]	0.94 [0.93, 0.94]	0.97 [0.96, 0.97]
**HM-POS5**	0.39 (0.06)	7 (34)	[-60, 74]	28	0.84 [0.81, 0.86]	0.91 [0.89, 0.93]	0.84 [0.82, 0.86]	0.92 [0.90, 0.93]
**HM-POS4**	0.38 (0.06)	−7 (35)	[-75, 61]	27	0.83 [0.80, 0.85]	0.91 [0.89, 0.92]	0.83 [0.81, 0.86]	0.91 [0.90, 0.92]
**HM-POS3**	0.36 (0.06)	−24 (35)	[-92, 44]	33	0.76 [0.49, 0.87]	0.87 [0.65, 0.93]	0.82 [0.80, 0.85]	0.90 [0.89, 0.92]
**HM-VEL100**	0.40 (0.07)	20 (32)	[-43, 83]	33	0.84 [0.66, 0.91]	0.91 [0.79, 0.95]	0.88 [0.86, 0.89]	0.93 [0.92, 0.94]
**HM-VEL80**	0.39 (0.07)	5 (38)	[-70, 80]	29	0.83 [0.81, 0.86]	0.91 [0.89, 0.92]	0.84 [0.81, 0.86]	0.91 [0.90, 0.92]
**HM-VEL50**	0.35 (0.08)	−33 (52)	[-54, 131]	42	0.66 [0.40, 0.79]	0.80 [0.57, 0.88]	0.73 [0.69, 0.76]	0.84 [0.82, 0.87]
**IMU-ACC**	0.41 (0.06)	30 (24)	[-17, 76]	32	0.83 [0.13, 0.94]	0.91 [0.22, 0.97]	0.93 [0.91, 0.94]	0.96 [0.95, 0.97]
**IMU-JERK**	0.37 (0.07)	−11 (29)	[-67, 45]	24	0.88 [0.83, 0.92]	0.94 [0.91, 0.96]	0.90 [0.88, 0.91]	0.95 [0.94, 0.95]
**FP-ZC**	0.35 (0.05)	−34 (43)	[-118, 51]	45	0.59 [0.20, 0.76]	0.74 [0.34, 0.87]	0.70 [0.65, 0.73]	0.82 [0.79, 0.84]

## 4 Discussion

The focus in our study was on comparing the HR detection results in the heel motion pattern. Additionally, we assessed the consistency and precision of these methods across different scenarios (Fig. 1, Table 2) and in comparison to visually detected HR (Table 3, Fig. 3). Our primary objective was to identify the most suitable point during heel rise for precisely separating the mid-stance and terminal stance phases. Simultaneously, our secondary objective involved developing and validating an IMU-based method for HR detection.

The initial HR detection method used a threshold for the heel's vertical position, similar to traditional footswitch-based HR detection. However, our analysis revealed the significant impact of subtle individual heel rising, termed ‘pre-rising,’ between MST and RIA on methods relying on positional and velocity data. This pre-rising led to earlier HR detection by position and velocity-based methods, introducing inconsistency in identifying the same point in the gait motion pattern (Fig. 2 and Fig. S6). The pre-rising, when occurring, was shown to begin right after MST, when the swinging limb advances anterior to stance limb and the center of pressure moves on the forefoot. We suspect that pre-rising may be caused by the individual differences in calf muscle activity prior to full forefoot rocker motion. These methods were also influenced by footwear conditions, resulting in relatively later HR detection and increased variability with shoes (Table 2). For users of position-based methods or similar approaches, we recommend favoring barefoot walking to minimize variability in detected HR. Additionally, velocity-based methods, as proposed in Refs. [14,16,20], were notably sensitive to speed, detecting HR much earlier in fast-paced scenarios (Table 2, Fig. 1, Fig. 3).

The force plate-based FP-ZC method, relying on anteroposterior force transition (Fig. 1e), consistently identified the acceleration trough before RIA (Fig. 1c) when considering the mean detection point across participants. However, individual subject and stride analyses revealed variability in this alignment (Fig. 3 and Fig. S6), along with the high variability within a leg across scenarios (Table 2), highlighting the subtle and variable nature of this force transition. Furthermore, FP-ZC displayed insensitivity to footwear differences and speed, except for a minor change at high speeds (Table 2), and low agreement with visually detected HR (Table 3, Fig. 3). This suggests that while FP-ZC is suitable for distinguishing mid-stance and terminal stance phases on force plates, it may not directly represent kinematic-based HR, instead reflecting more on muscle control and kinetics.

Acceleration and jerk-based methods, HM-ACC and HM-JERK, designed to identify the RIA, demonstrated robustness against variations in pre-rising and consistently identified the same moment in the heel marker's motion pattern (Fig. 1a–c, Fig. S6), showing high agreement with visually detected HR (Table 3, Fig. 3). Their mean vertical position remained relatively stable across scenarios, indicating robust event detection. However, HM-ACC occasionally detected HR events prematurely at high speeds, especially in cases with significant pre-rising (Fig. S5), leading to increased variability with both HM-ACC and HM-JERK in fast-paced scenarios. Incorporating a speed adjustment into the algorithm could address this issue, which may also be necessary for lower speeds and pathological gait.

HM-ACC otherwise showed notably lower variability of cycle point compared to all other methods (Table 2) but tended to identify HR significantly after RIA. In contrast, HM-JERK consistently captured an event closely aligned with the RIA across scenarios (Fig. 1, Fig. 3), where the progressing COM and swinging limb inertia initiate the accelerating rise of the heel. Additionally, HM-JERK exhibited low variability in normal and slow-speed conditions (Table 2). In context, literature values [14,19] aligned well with the mean cycle point values of HM-JERK at a normal speed, further supporting its suitability for HR detection.

While other methods may have specific purposes—such as recognizing heel rise as a distinct phase (from MST to HM-ACC), finding the transition from braking to accelerating (FP-ZC), or aiming to replicate results similar to footswitches (HM-POS)—HM-JERK stands out as a robust solution with many strengths. It detects the HR event across scenarios in a consistent manner, signifying the onset of rapid vertical acceleration of the heel, making it well-suited for separating gait phases.

Additionally, the tested methods, IMU-ACC and IMU-JERK, closely mirrored results obtained using the optical motion capture-based HM-ACC and HM-JERK methods and visually detected HR (Table 2, Table 3, Fig. 1, Fig. 3). While the IMU's motion is less affected by heel rise compared to the heel marker due to the ankle's dorsiflexed position at the time of HR, the effect is still significant enough to use similar detection methods, albeit with a lower threshold. Consequently, the accuracy of our IMU-based method for detecting RIA during the heel rise process is commendable.

Finally, it is important to note the effect of shoe type, particularly the sole's elasticity, on heel rise motion. In this study, participants used their own non-rigid indoor sports shoes, which varied by model and could impact heel rise motion. Walking with more rigid shoes would likely have an even more significant effect [12]. However, the differences in heel rise motion patterns between barefoot and shod walking varied among participants. For some, the patterns remained highly similar, while for others, HR occurred significantly earlier in barefoot walking (Fig. S6), which emphasizes the individual nature of heel rise motion.

Our study has some limitations. We did not use pressure insoles or footswitches, missing insights into HR detection. The reference method, visual detection, is subjective and identifies the moment the heel begins its rapid rise, which may differ from pressure-based HR detection. However, the mean values of visually detected HR align closely with literature values for pressure-based HR. As the heel rise motion varies among subjects, a more diverse study population would have enhanced reliability, despite a high number of strides measured. Additionally, our findings primarily apply to healthy subjects and may require further validation to fully represent different pathological gait models. Nonetheless, our study's various scenarios demonstrate competence in capturing diverse heel rise strategies, which can later be validated for wider population.

## 5 Conclusion

In conclusion, our study provides valuable insights into the complexities of heel rise event detection, including the critical influence of pre-rising. While various methods exhibit strengths and limitations, the HM-JERK method emerges as a robust and consistent approach for heel rise detection. Additionally, our shank-attached IMU-based methods closely align with OMC-based methods, promising an accurate and practical alternative for heel rise detection. Future research should focus on refining these methods and expanding their application to a wider range of gait models.

## CRediT authorship contribution statement

**Mikko Salminen:** Writing – original draft, Software, Methodology, Investigation, Conceptualization. **Jarmo Perttunen:** Writing – review & editing, Methodology, Conceptualization. **Janne Avela:** Writing – review & editing, Resources, Methodology, Conceptualization. **Antti Vehkaoja:** Writing – review & editing, Supervision, Resources, Methodology, Conceptualization.

## Declaration of generative AI and AI-assisted technologies in the writing process

During the preparation of this work the original draft's author used ChatGPT 3.5 and Trinka AI to improve readability, language, and grammar. After using these tools for enhancement, the author carefully reviewed and edited the content as needed and takes full responsibility for the content of the publication.

## Declaration of competing interest

The authors declare that they have no known competing financial interests or personal relationships that could have appeared to influence the work reported in this paper.
