# Supplementary material for: Comparative assessment of heel rise detection for consistent gait phase separation
Source: Heliyon. 2024 Jun 24;10(13):e33546. doi: 10.1016/j.heliyon.2024.e33546 (PMC11260980; doi:10.1016/j.heliyon.2024.e33546)
Supplement: Multimedia component 1 [file mmc1.docx]

**SUPPLEMENTARY MATERIAL**


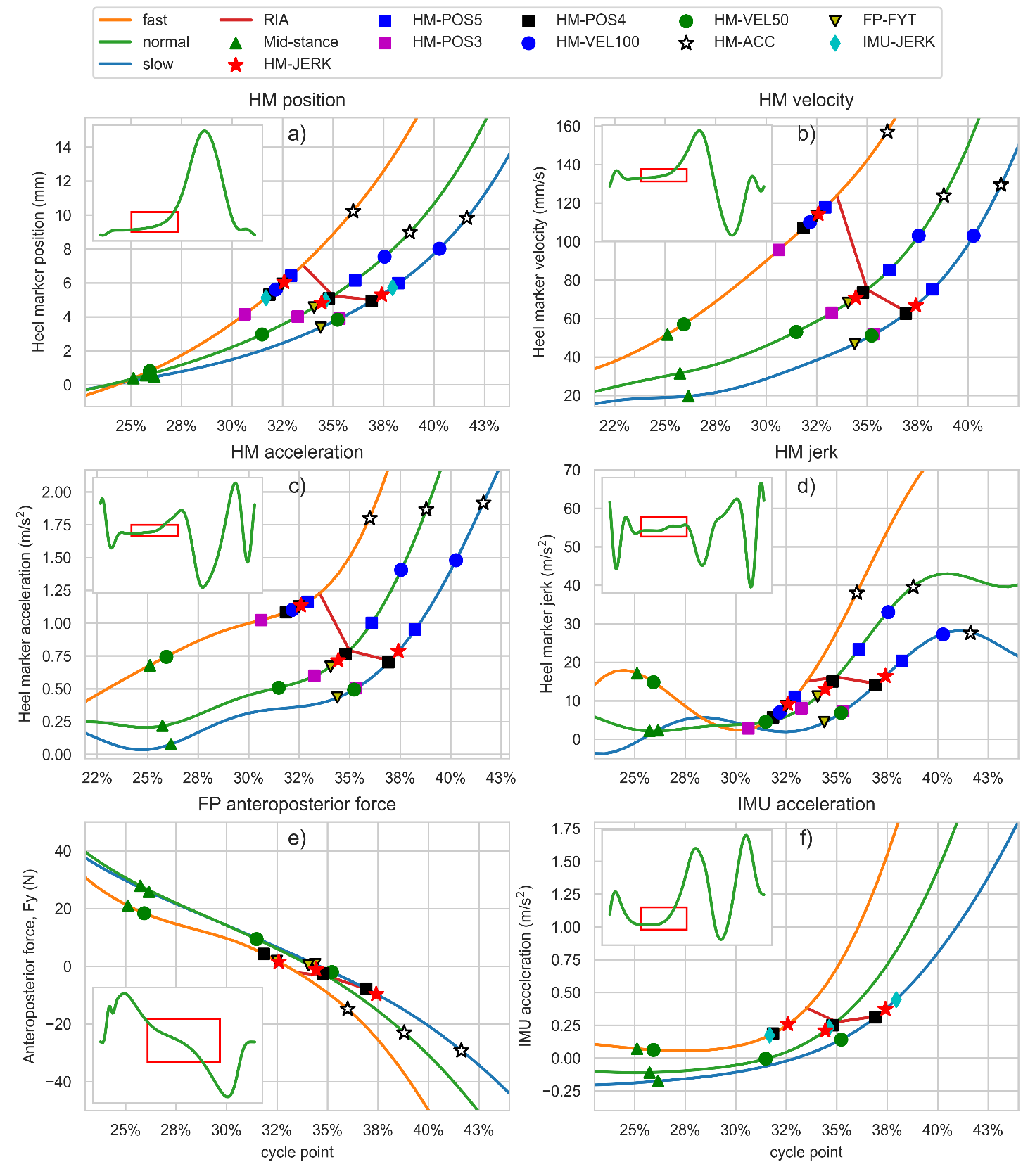
Fig. S1. Mean signal plots during heel rise for fast (orange), normal (green) and slow (blue) for barefoot walking, along with mean HR detection points of different methods: a) Heel marker vertical position relative to position at MST, b) velocity, c) acceleration, d) jerk (velocity of acceleration), e) force plate anteroposterior force (Fy), f) IMU longitudinal acceleration (gravity being subtracted). The red line indicates the RIA point, which we have visually detected based on these mean velocity and acceleration plot shapes. Smaller subplots depict signal shape during the entire gait cycle.


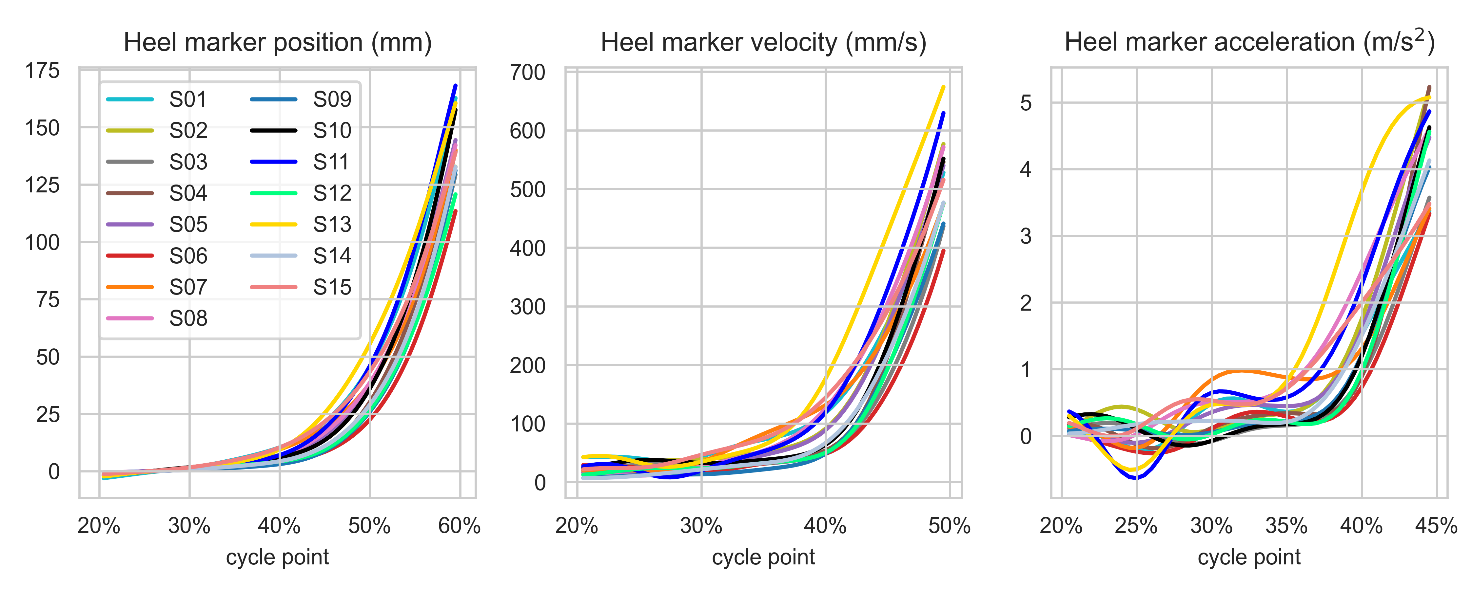


Fig. S2. Mean vertical position of the heel marker relative to its position at MST, along with velocity and acceleration. The figure includes mean line plots for each subject walking in shoes at a normal speed.


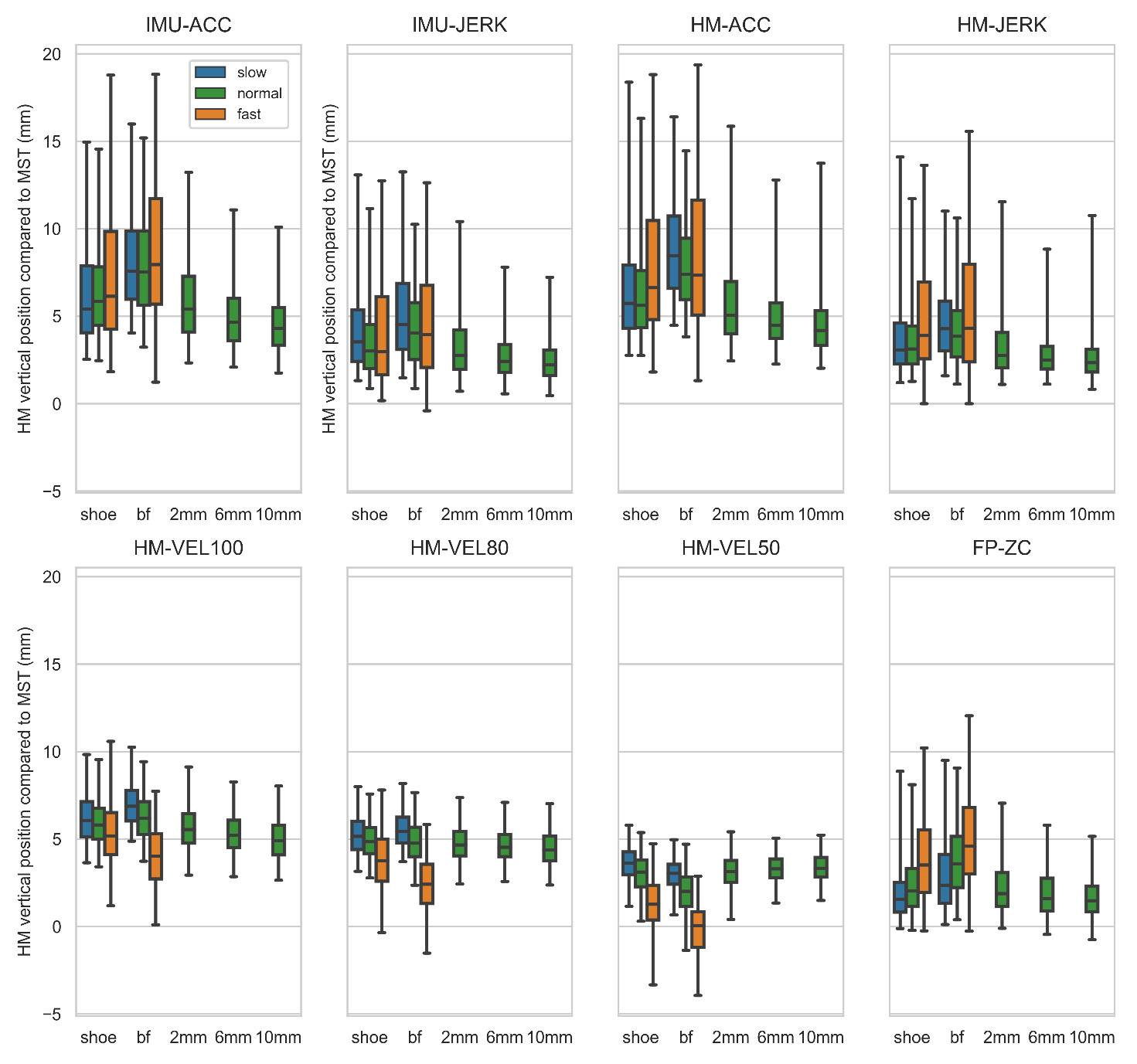


Fig. S3. Heel markers vertical position compared to position at MST. Shod and barefoot walking with slow, normal, and fast speed.


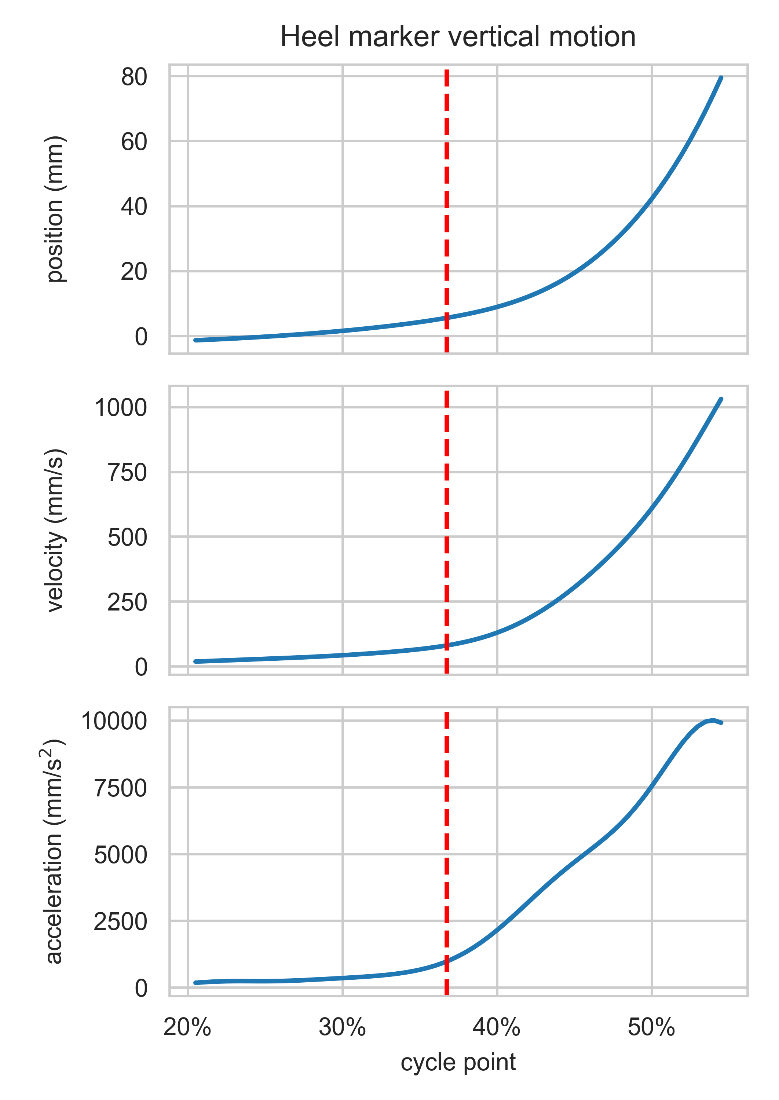


Fig. S4. Mean point of visually detected HR on the mean heel marker position, velocity, and acceleration plots.


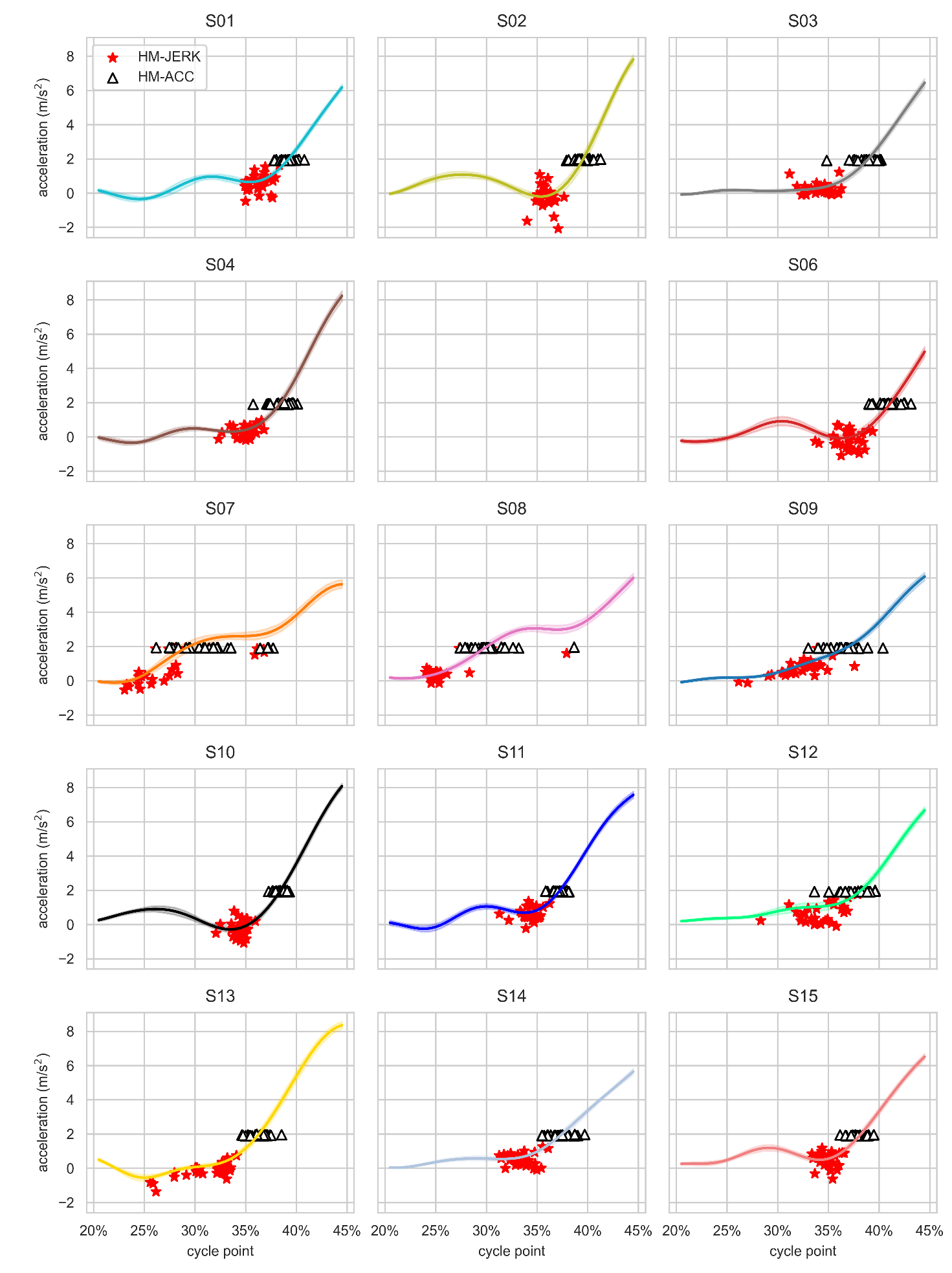


Fig. S5. Alignment of the individual HR events detected with HM-JERK and HM-ACC methods to the heel marker’s mean vertical acceleration pattern. The data consist of the left leg cycles while walking in shoes at fast speed. Shaded areas indicate 95% confidence intervals.


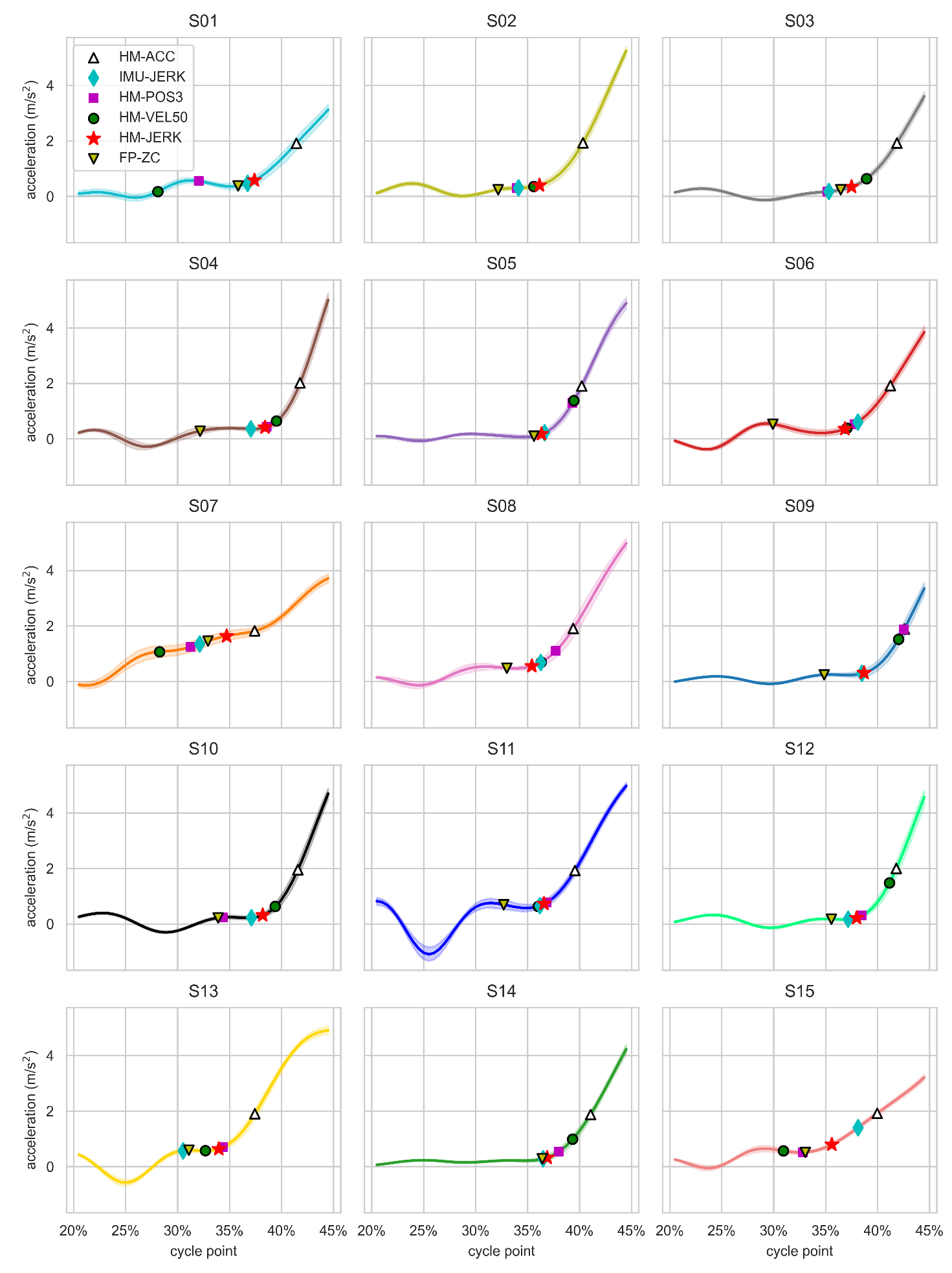


Fig.S6. Alignment of HR detection methods to the heel marker’s vertical acceleration pattern while walking in shoes at normal speed. Shaded areas indicate 95% confidence intervals of the mean. The figure data contains only the force plate strides of the left leg.


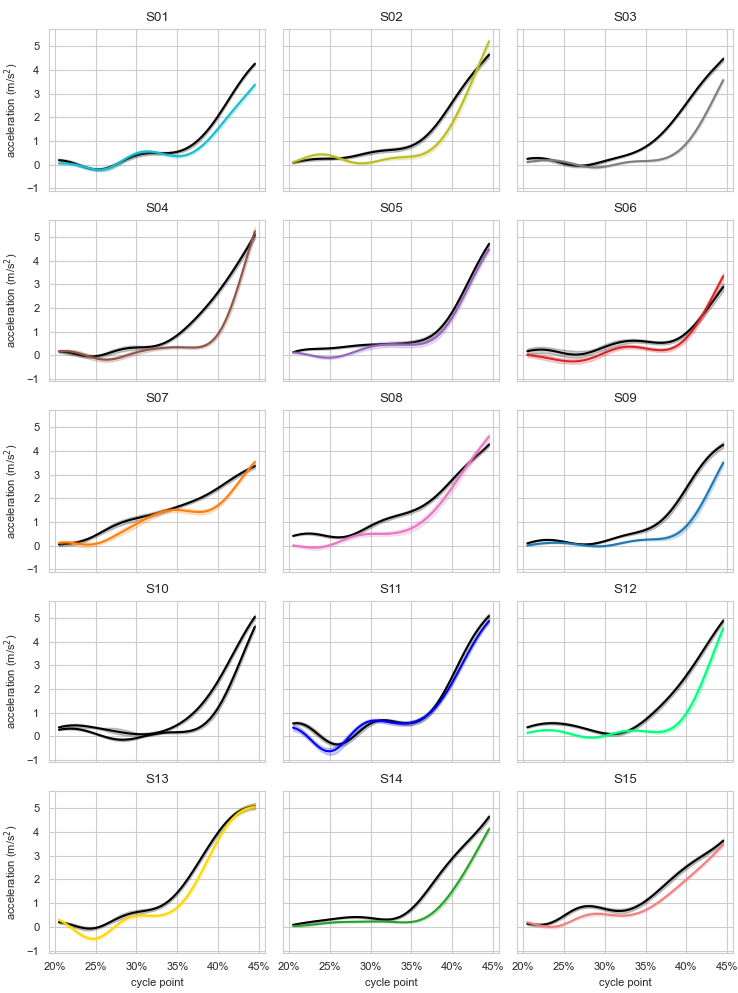


Figure S7. Comparison of vertical acceleration patterns of the heel marker during barefoot walking (black line) and shod walking (colored line). The data encompasses all gait cycles at normal walking speed for both feet.

Table S1. Data Normality by Speed & Shoe Type. Table describes the normality of leg-specific mean values across speed & shoe-specific scenarios (left side of the table) and the normality of detected events within leg, speed, & shoe type-specific scenarios (right side of the table). The normality rate indicates the percentage of leg/scenario combinations where data is normally distributed according to the Shapiro-Wilk test.

|  |  | **Speed & shoe type-specific data subsets** | | | | | | | |  | **Leg, speed & shoe type-specific data subsets** | |
| --- | --- | --- | --- | --- | --- | --- | --- | --- | --- | --- | --- | --- |
|  | Speed | fast | | slow | | normal | | Normality rate | Mean p-value (Shapiro-Wilk) |  | Normality rate | Mean p-value (Shapiro-Wilk) |
|  | Shoe | bf | shoe | bf | shoe | bf | shoe |  |  |  |  |  |
| MST | Normality | FALSE | TRUE | TRUE | FALSE | TRUE | TRUE | 67 % | 0.34 |  | 89 % | 0.46 |
|  | p-value | 4 % | 10 % | 62 % | 4 % | 65 % | 60 % |  |  |  |  |  |
| HM-ACC | Normality | TRUE | TRUE | TRUE | FALSE | TRUE | FALSE | 67 % | 0.40 |  | 87 % | 0.42 |
|  | p-value | 94 % | 30 % | 57 % | 2 % | 52 % | 4 % |  |  |  |  |  |
| HM-JERK | Normality | TRUE | TRUE | TRUE | TRUE | TRUE | TRUE | 100 % | 0.40 |  | 68 % | 0.29 |
|  | p-value | 28 % | 18 % | 94 % | 57 % | 39 % | 6 % |  |  |  |  |  |
| HM-VEL100 | Normality | TRUE | TRUE | TRUE | TRUE | FALSE | FALSE | 67 % | 0.23 |  | 71 % | 0.30 |
|  | p-value | 13 % | 26 % | 93 % | 6 % | 2 % | 1 % |  |  |  |  |  |
| HM-VEL50 | Normality | TRUE | TRUE | TRUE | FALSE | TRUE | FALSE | 67 % | 0.40 |  | 57 % | 0.21 |
|  | p-value | 97 % | 8 % | 45 % | 1 % | 88 % | 3 % |  |  |  |  |  |
| HM-VEL80 | Normality | TRUE | TRUE | TRUE | FALSE | FALSE | FALSE | 50 % | 0.24 |  | 65 % | 0.28 |
|  | p-value | 20 % | 26 % | 84 % | 4 % | 5 % | 4 % |  |  |  |  |  |
| HM-POS5 | Normality | TRUE | TRUE | TRUE | TRUE | TRUE | TRUE | 100 % | 0.60 |  | 81 % | 0.37 |
|  | p-value | 81 % | 42 % | 87 % | 18 % | 92 % | 39 % |  |  |  |  |  |
| HM-POS3 | Normality | TRUE | TRUE | TRUE | TRUE | TRUE | TRUE | 100 % | 0.67 |  | 81 % | 0.38 |
|  | p-value | 95 % | 47 % | 96 % | 49 % | 85 % | 31 % |  |  |  |  |  |
| HM-POS4 | Normality | TRUE | TRUE | TRUE | TRUE | TRUE | TRUE | 100 % | 0.63 |  | 84 % | 0.39 |
|  | p-value | 81 % | 49 % | 96 % | 30 % | 86 % | 38 % |  |  |  |  |  |
| IMU-ACC | Normality | TRUE | TRUE | TRUE | TRUE | TRUE | TRUE | 100 % | 0.29 |  | 63 % | 0.29 |
|  | p-value | 27 % | 29 % | 15 % | 16 % | 52 % | 33 % |  |  |  |  |  |
| IMU-JERK | Normality | TRUE | TRUE | FALSE | TRUE | TRUE | TRUE | 83 % | 0.40 |  | 69 % | 0.29 |
|  | p-value | 98 % | 35 % | 5 % | 8 % | 77 % | 18 % |  |  |  |  |  |
| **ALL METHODS** |  |  |  |  |  |  |  | **82 %** | **0.42** |  | **74 %** | **0.34** |

Table S2. Effect size calculation. Effect sizes (Cohen's d) are calculated between HR detection method results. The presented numbers show the mean absolute values of Cohen's d effect size calculations for different method-method combinations across various shoe (barefoot & shoe) and speed (fast, normal, slow) scenarios.

| **Detection method** | **HM-ACC** | **HM-JERK** | **HM-VEL100** | **HM-VEL50** | **HM-VEL80** | **HM-POS5** | **HM-POS3** | **HM-POS4** | **IMU-ACC** | **IMU-JERK** |
| --- | --- | --- | --- | --- | --- | --- | --- | --- | --- | --- |
| **HM-ACC** |  | 1,86 | 0,56 | 2,14 | 1,10 | 1,06 | 2,30 | 1,60 | 0,16 | 1,79 |
| **HM-JERK** | 1,86 |  | 1,09 | 0,83 | 0,70 | 0,59 | 0,52 | 0,25 | 1,54 | 0,24 |
| **HM-VEL100** | 0,56 | 1,09 |  | 1,51 | 0,50 | 0,47 | 1,42 | 0,84 | 0,43 | 1,02 |
| **HM-VEL50** | 2,14 | 0,83 | 1,51 |  | 1,04 | 1,27 | 0,57 | 0,89 | 1,91 | 0,73 |
| **HM-VEL80** | 1,10 | 0,70 | 0,50 | 1,04 |  | 0,30 | 0,89 | 0,49 | 0,89 | 0,61 |
| **HM-POS5** | 1,06 | 0,59 | 0,47 | 1,27 | 0,30 |  | 1,08 | 0,48 | 0,82 | 0,62 |
| **HM-POS3** | 2,30 | 0,52 | 1,42 | 0,57 | 0,89 | 1,08 |  | 0,59 | 1,95 | 0,43 |
| **HM-POS4** | 1,60 | 0,25 | 0,84 | 0,89 | 0,49 | 0,48 | 0,59 |  | 1,32 | 0,28 |
| **IMU-ACC** | 0,16 | 1,54 | 0,43 | 1,91 | 0,89 | 0,82 | 1,95 | 1,32 |  | 1,51 |
| **IMU-JERK** | 1,79 | 0,24 | 1,02 | 0,73 | 0,61 | 0,62 | 0,43 | 0,28 | 1,51 |  |
